# 2-(4-*tert*-Butyl­phen­yl)-1*H*-imidazo[4,5-*f*][1,10]phenanthroline sesquihydrate

**DOI:** 10.1107/S1600536809020261

**Published:** 2009-06-06

**Authors:** Chun-Yang Zheng, Ting-Quan Sun

**Affiliations:** a Hubei Key Laboratory of Pollutant Analysis and Reuse Technology, Hubei Normal University, Huangshi 435002, People’s Republic of China

## Abstract

In the title compound, C_23_H_20_N_4_·1.5H_2_O, the mean planes of the imidazo[4,5-*f*][1,10]phenanthroline system and the benzene ring make a dihedral angle of 21.76 (2)°. One water O atom lies on a twofold rotation axis. The organic mol­ecules and water mol­ecules are linked *via* N—H⋯O, O—H⋯N and O—H⋯O hydrogen bonds. Weak inter­molecular C—H⋯N hydrogen bonds and π–π stacking inter­actions between inversion-related phenanthroline rings complete the three-dimensional hydrogen-bonding network in the crystal structure. The stacking distance is short at 3.513 (2) Å and the perpendicular distance between the rings is 3.355 Å. The three methyl groups are disordered over two positions, with a site-occupancy ratio of 0.875 (14):0.125 (14).

## Related literature

For 1,10-phenanthroline derivatives as ligands, see: Cardinaels *et al.* (2005 ▶); Liu *et al.* (2005 ▶). For the crystal structures of 1,10-phenanthroline derivatives, see: Bian *et al.* (2002 ▶); Wu *et al.* (1998 ▶). For aromatic π–π stacking inter­actions, see: Janiak (2000 ▶).
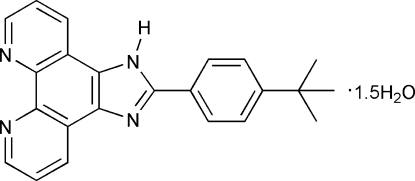


## Experimental

### 

#### Crystal data

C_23_H_20_N_4_·1.5H_2_O
*M* *_r_* = 379.46
Tetragonal, 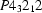

*a* = 14.809 (4) Å
*c* = 18.281 (6) Å
*V* = 4009.1 (19) Å^3^
*Z* = 8
Mo *K*α radiation
μ = 0.08 mm^−1^
*T* = 298 K
0.16 × 0.13 × 0.10 mm

#### Data collection

Bruker SMART CCD area-detector diffractometer
Absorption correction: none
46858 measured reflections
2670 independent reflections
2508 reflections with *I* > 2σ(*I*)
*R*_int_ = 0.054

#### Refinement

*R*[*F* ^2^ > 2σ(*F* ^2^)] = 0.068
*wR*(*F* ^2^) = 0.167
*S* = 1.28
2670 reflections
304 parameters
53 restraints
H atoms treated by a mixture of independent and constrained refinement
Δρ_max_ = 0.30 e Å^−3^
Δρ_min_ = −0.15 e Å^−3^

### 

Data collection: *SMART* (Bruker, 1997 ▶); cell refinement: *SAINT* (Bruker, 1999 ▶); data reduction: *SAINT*; program(s) used to solve structure: *SHELXS97* (Sheldrick, 2008 ▶); program(s) used to refine structure: *SHELXL97* (Sheldrick, 2008 ▶); molecular graphics: *SHELXTL* (Sheldrick, 2008 ▶); software used to prepare material for publication: *SHELXTL* and *PLATON* (Spek, 2009 ▶).

## Supplementary Material

Crystal structure: contains datablocks global, I. DOI: 10.1107/S1600536809020261/si2177sup1.cif

Structure factors: contains datablocks I. DOI: 10.1107/S1600536809020261/si2177Isup2.hkl

Additional supplementary materials:  crystallographic information; 3D view; checkCIF report

## Figures and Tables

**Table 1:** Hydrogen-bond geometry (Å, °)

*D*—H⋯*A*	*D*—H	H⋯*A*	*D*⋯*A*	*D*—H⋯*A*
O1—H1*A*⋯N1^i^	0.83 (3)	2.45 (3)	3.193 (5)	151 (5)
O1—H1*A*⋯N2^i^	0.83 (3)	2.19 (4)	2.853 (4)	137 (5)
O1—H1*B*⋯O2^ii^	0.83 (3)	2.065 (16)	2.878 (3)	168 (5)
O2—H2*A*⋯N4	0.82 (3)	2.15 (2)	2.914 (4)	156 (5)
N3—H3⋯O1	0.86 (3)	1.90 (3)	2.754 (4)	176 (4)
C12—H12⋯N4^iii^	0.93	2.53	3.346 (4)	147

Symmetry codes: (i) 
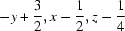
; (ii) 
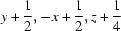
; (iii) 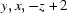.

**Fig. 1.**
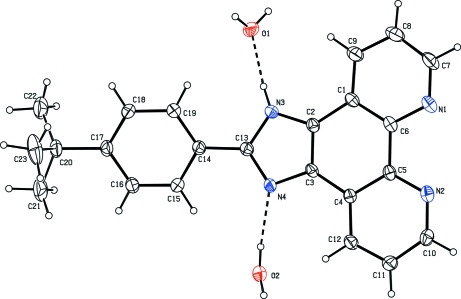
View of the molecular structure of (I), showing the atomic numbering. Displacement ellipsoids are drawn at the 50% probability level. Hydrogen bonds are indicated by dashed lines. The three disordered methyl groups were omitted for clarity.

**Fig. 2.**
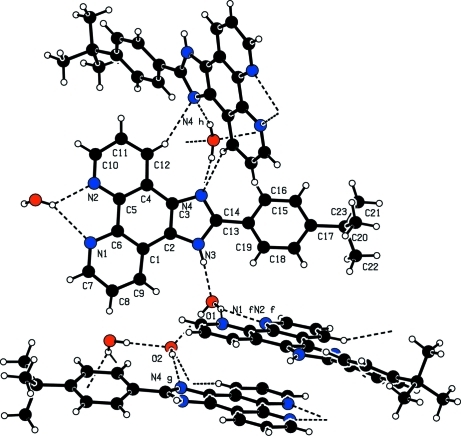
A section of the structure of (I) with intermolecular hydrogen bonds indicated as dashed lines.

## supplementary crystallographic information

### Comment

1,10-Phenanthroline and its derivatives are commonly used as ligands in metal complexes (Bian *et al.* 2002; Cardinaels *et al.* 2005; Liu *et al.* 2005; Wu *et al.* 1998). We report here the structure of the title compound (I) (Fig. 1), which was synthesized from [4,5-*f*]1,10-phenanthroline. The mean plane of the [4,5-*f*]1,10-phenanthroline moiety and the benzene ring (C14 - C19) make a dihedral angle of 21.76 (2) °. The water oxygen atom O2 occupies a twofold rotation axis, generating a symmetric four-centre hydrogen bond system that link two imidazo groups and two water molecules O1 *via* O—H···N and O—H···O bonds (Fig. 2). The other water molecules O1 link the two phenanthroline N-atoms through bifurcated O—H···N hydrogen bonds (Table 1). Intermolecular C—H···N hydrogen bonds and π—π stacking interactions (Janiak, 2000) between inversion related phenanthroline rings complete the hydrogen bonding network in the crystal structure. The short stacking distance Cg*_i_*—Cg*_j_* is 3.513 (2) Å, the perpendicular distance between the rings is 3.355 Å, and the dihedral angle between the rings is 2.48 °. Cg is the centroid of ring (N2, C5, C4, C12, C11, C10), a symmetry code for Cg*_j_* was given as (1 - *y*, 1 - *x*, 5/2 - *z*. The three methyl groups are disordered over two positions, with a site occupancy ratio of *ca* 7:1.

### Experimental

1,10-Phenanthroline-5,6-dione (0.84 g, 0.004 mol) and ammonium acetate (3.1 g, 0.04 mmol) were dissolved in 40 ml of hot glacial acetic acid. While the mixture was stirred, a solution of 4-*tert*-butylbenzaldehyde (0.65 g, 0.004 mmol) in 10 ml of glacial acetic acid was added dropwise to the mixture. The mixture was heated at 363 K for 3 h and was then poured in 200 ml of water. The solution was neutralized with ammonia to pH=8 and was then cooled to room temperature. The precipitate was filtered off and recrystallized from dillute ethanol solution to give the title compound (I). Crystals suitable for X-ray diffraction were grown by slow evaporation of the EtOH solutions at room temperature.

### Refinement

The methyl groups were found to be disordered over two orientations. The occupancies of the disordered positions C21/C21', C22/C22' and C23/C23' were refined to 0.875 (14)/0.125 (14). Suitable restraints were applied to the C—C distances involving the disordered atoms. The methyl H atoms were constrained to an ideal geometry with C—H distances of 0.96 Å and *U*_iso_(H) = 1.5 *U*_eq_(C), but each group was allowed to rotate freely about its C—C bond. Other H atoms were placed in geometrically idealized positions and constrained to ride on their parent C atoms, with C—H distances of 0.93 to 0.97 Å, and with *U*_iso_(H) = 1.2 *U*_eq_(C). All H atoms on N atoms were positioned geometrically and refined as riding atoms, with N—H = 0.86 Å and *U*_iso_(H) = 1.2 *U*_eq_(N). The H atoms of the waters were located in a Fourier map following isotropic refinement. In the absence of significant anomalous scattering effects, Friedel related intensity reflections were averaged.

### Crystal data

**Table:**

C_23_H_20_N_4_·1.5H_2_O	*D*_x_ = 1.257 Mg m^−^^3^
*M**_r_* = 379.46	Mo *K*α radiation, λ = 0.71073 Å
Tetragonal, *P*4_3_2_1_2	Cell parameters from 5405 reflections
Hall symbol: P4nw 2abw	θ = 2.2–22.5°
*a* = 14.809 (4) Å	µ = 0.08 mm^−^^1^
*c* = 18.281 (6) Å	*T* = 298 K
*V* = 4009.1 (19) Å^3^	Block, yellow
*Z* = 8	0.16 × 0.13 × 0.10 mm
*F*(000) = 1608	

### Data collection

**Table:**

Bruker SMART CCD area-detector diffractometer	2508 reflections with *I* > 2σ(*I*)
Radiation source: fine-focus sealed tube	*R*_int_ = 0.054
graphite	θ_max_ = 27.5°, θ_min_ = 1.9°
φ and ω scans	*h* = −19→19
46858 measured reflections	*k* = −19→19
2670 independent reflections	*l* = −23→23

### Refinement

**Table:**

Refinement on *F*^2^	Primary atom site location: structure-invariant direct methods
Least-squares matrix: full	Secondary atom site location: difference Fourier map
*R*[*F*^2^ > 2σ(*F*^2^)] = 0.068	Hydrogen site location: inferred from neighbouring sites
*wR*(*F*^2^) = 0.167	H atoms treated by a mixture of independent and constrained refinement
*S* = 1.28	*w* = 1/[σ^2^(*F*_o_^2^) + (0.0765*P*)^2^ + 0.9688*P*] where *P* = (*F*_o_^2^ + 2*F*_c_^2^)/3
2670 reflections	(Δ/σ)_max_ < 0.001
304 parameters	Δρ_max_ = 0.30 e Å^−^^3^
53 restraints	Δρ_min_ = −0.15 e Å^−^^3^

### Special details

**Table:**

Geometry. All e.s.d.'s (except the e.s.d. in the dihedral angle between two l.s. planes) are estimated using the full covariance matrix. The cell e.s.d.'s are taken into account individually in the estimation of e.s.d.'s in distances, angles and torsion angles; correlations between e.s.d.'s in cell parameters are only used when they are defined by crystal symmetry. An approximate (isotropic) treatment of cell e.s.d.'s is used for estimating e.s.d.'s involving l.s. planes.
Refinement. Refinement of *F*^2^ against ALL reflections. The weighted *R*-factor *wR* and goodness of fit *S* are based on *F*^2^, conventional *R*-factors *R* are based on *F*, with *F* set to zero for negative *F*^2^. The threshold expression of *F*^2^ > σ(*F*^2^) is used only for calculating *R*-factors(gt) *etc*. and is not relevant to the choice of reflections for refinement. *R*-factors based on *F*^2^ are statistically about twice as large as those based on *F*, and *R*- factors based on ALL data will be even larger.

### Fractional atomic coordinates and isotropic or equivalent isotropic displacement parameters (Å^2^)

**Table:**

	*x*	*y*	*z*	*U*_iso_*/*U*_eq_	Occ. (<1)
C1	0.7310 (2)	0.4019 (2)	1.19064 (17)	0.0436 (7)	
C2	0.6832 (2)	0.3733 (2)	1.12626 (16)	0.0412 (7)	
C3	0.6074 (2)	0.4156 (2)	1.10102 (16)	0.0392 (7)	
C4	0.5709 (2)	0.4934 (2)	1.13671 (16)	0.0399 (7)	
C5	0.6151 (2)	0.5242 (2)	1.20079 (17)	0.0424 (7)	
C6	0.6959 (2)	0.4778 (2)	1.22760 (17)	0.0453 (8)	
N1	0.7345 (2)	0.5111 (2)	1.28912 (17)	0.0593 (9)	
C7	0.8061 (3)	0.4692 (3)	1.3142 (2)	0.0723 (13)	
H7	0.8325	0.4915	1.3567	0.087*	
C8	0.8453 (3)	0.3944 (4)	1.2822 (3)	0.0760 (14)	
H8	0.8961	0.3676	1.3027	0.091*	
C9	0.8074 (3)	0.3605 (3)	1.2194 (2)	0.0623 (10)	
H9	0.8325	0.3104	1.1965	0.075*	
N2	0.5840 (2)	0.5952 (2)	1.23928 (16)	0.0505 (7)	
C10	0.5114 (3)	0.6373 (3)	1.2156 (2)	0.0538 (9)	
H10	0.4901	0.6860	1.2428	0.065*	
C11	0.4647 (3)	0.6138 (3)	1.1531 (2)	0.0569 (9)	
H11	0.4139	0.6460	1.1385	0.068*	
C12	0.4950 (2)	0.5420 (2)	1.11298 (19)	0.0504 (8)	
H12	0.4654	0.5254	1.0702	0.060*	
C13	0.6348 (2)	0.3069 (2)	1.02710 (16)	0.0416 (7)	
C14	0.6341 (2)	0.2461 (2)	0.96339 (16)	0.0426 (7)	
C15	0.5563 (2)	0.2359 (2)	0.92178 (18)	0.0480 (8)	
H15	0.5038	0.2657	0.9359	0.058*	
C16	0.5553 (3)	0.1825 (2)	0.85987 (18)	0.0500 (8)	
H16	0.5020	0.1764	0.8335	0.060*	
C17	0.6319 (3)	0.1377 (2)	0.83626 (18)	0.0510 (9)	
C18	0.7096 (3)	0.1491 (3)	0.8774 (2)	0.0616 (11)	
H18	0.7623	0.1202	0.8627	0.074*	
C19	0.7112 (3)	0.2021 (3)	0.93992 (19)	0.0566 (10)	
H19	0.7646	0.2082	0.9663	0.068*	
C20	0.6326 (3)	0.0806 (3)	0.76548 (19)	0.0646 (11)	
C21	0.5375 (4)	0.0646 (6)	0.7367 (4)	0.110 (3)	0.875 (14)
H21A	0.5404	0.0291	0.6928	0.165*	0.875 (14)
H21B	0.5094	0.1216	0.7262	0.165*	0.875 (14)
H21C	0.5028	0.0331	0.7729	0.165*	0.875 (14)
C22	0.6769 (8)	−0.0099 (5)	0.7785 (4)	0.122 (4)	0.875 (14)
H22A	0.6722	−0.0460	0.7351	0.183*	0.875 (14)
H22B	0.6472	−0.0401	0.8182	0.183*	0.875 (14)
H22C	0.7394	−0.0010	0.7904	0.183*	0.875 (14)
C23	0.6840 (7)	0.1327 (6)	0.7075 (3)	0.125 (4)	0.875 (14)
H23A	0.7410	0.1519	0.7269	0.188*	0.875 (14)
H23B	0.6495	0.1846	0.6929	0.188*	0.875 (14)
H23C	0.6940	0.0945	0.6658	0.188*	0.875 (14)
C21'	0.565 (3)	0.003 (3)	0.765 (3)	0.13 (2)	0.125 (14)
H21D	0.5711	−0.0303	0.7199	0.188*	0.125 (14)
H21E	0.5047	0.0268	0.7680	0.188*	0.125 (14)
H21F	0.5762	−0.0362	0.8054	0.188*	0.125 (14)
C22'	0.7261 (17)	0.040 (4)	0.752 (3)	0.099 (17)	0.125 (14)
H22D	0.7216	−0.0080	0.7169	0.149*	0.125 (14)
H22E	0.7490	0.0154	0.7976	0.149*	0.125 (14)
H22F	0.7664	0.0853	0.7347	0.149*	0.125 (14)
C23'	0.612 (4)	0.140 (3)	0.6995 (18)	0.12 (2)	0.125 (14)
H23D	0.6390	0.1146	0.6565	0.174*	0.125 (14)
H23E	0.6365	0.1996	0.7076	0.174*	0.125 (14)
H23F	0.5479	0.1446	0.6929	0.174*	0.125 (14)
N3	0.70020 (19)	0.30431 (19)	1.07917 (14)	0.0433 (6)	
H3	0.734 (2)	0.2580 (17)	1.086 (2)	0.052*	
N4	0.57632 (19)	0.37297 (18)	1.03858 (13)	0.0417 (6)	
O1	0.8076 (2)	0.1575 (2)	1.11023 (13)	0.0661 (9)	
H1A	0.852 (2)	0.159 (4)	1.0831 (19)	0.099*	
H1B	0.824 (3)	0.140 (4)	1.1511 (13)	0.099*	
O2	0.38586 (17)	0.38586 (17)	1.0000	0.0487 (8)	
H2A	0.4347 (17)	0.366 (3)	1.014 (3)	0.073*	

### Atomic displacement parameters (Å^2^)

**Table:**

	*U*^11^	*U*^22^	*U*^33^	*U*^12^	*U*^13^	*U*^23^
C1	0.0486 (18)	0.0470 (18)	0.0354 (14)	−0.0038 (15)	−0.0049 (13)	0.0075 (13)
C2	0.0490 (18)	0.0405 (16)	0.0341 (14)	−0.0017 (14)	−0.0012 (13)	0.0037 (12)
C3	0.0475 (17)	0.0393 (15)	0.0307 (13)	−0.0020 (13)	−0.0058 (12)	0.0041 (12)
C4	0.0453 (17)	0.0405 (16)	0.0340 (14)	−0.0022 (13)	−0.0008 (13)	0.0001 (12)
C5	0.0510 (18)	0.0420 (17)	0.0342 (14)	−0.0069 (14)	0.0002 (13)	0.0027 (12)
C6	0.0514 (18)	0.0490 (18)	0.0355 (15)	−0.0111 (15)	−0.0054 (14)	0.0018 (13)
N1	0.064 (2)	0.069 (2)	0.0451 (15)	−0.0063 (17)	−0.0158 (15)	−0.0029 (15)
C7	0.079 (3)	0.081 (3)	0.057 (2)	−0.006 (3)	−0.031 (2)	−0.004 (2)
C8	0.069 (3)	0.086 (3)	0.072 (3)	0.010 (3)	−0.031 (2)	0.002 (3)
C9	0.062 (2)	0.064 (2)	0.061 (2)	0.005 (2)	−0.0200 (19)	−0.0030 (19)
N2	0.0604 (18)	0.0486 (16)	0.0425 (14)	−0.0071 (13)	−0.0021 (14)	−0.0057 (13)
C10	0.061 (2)	0.0452 (18)	0.0552 (19)	−0.0009 (16)	0.0023 (18)	−0.0122 (17)
C11	0.060 (2)	0.048 (2)	0.062 (2)	0.0087 (17)	−0.0095 (18)	−0.0069 (17)
C12	0.058 (2)	0.0471 (18)	0.0459 (17)	0.0003 (16)	−0.0133 (16)	−0.0037 (15)
C13	0.0492 (17)	0.0409 (16)	0.0347 (14)	0.0019 (14)	−0.0003 (13)	0.0050 (13)
C14	0.0523 (18)	0.0408 (16)	0.0349 (14)	0.0042 (14)	−0.0006 (14)	0.0033 (13)
C15	0.0472 (19)	0.0515 (19)	0.0452 (16)	0.0083 (15)	0.0009 (15)	−0.0064 (15)
C16	0.058 (2)	0.051 (2)	0.0408 (16)	0.0000 (17)	−0.0041 (16)	−0.0042 (15)
C17	0.073 (2)	0.0489 (19)	0.0317 (14)	0.0097 (17)	0.0009 (16)	−0.0007 (14)
C18	0.066 (2)	0.074 (3)	0.0446 (18)	0.029 (2)	−0.0004 (18)	−0.0092 (18)
C19	0.056 (2)	0.069 (2)	0.0449 (18)	0.0161 (19)	−0.0064 (16)	−0.0076 (17)
C20	0.090 (3)	0.069 (3)	0.0340 (17)	0.016 (2)	−0.0018 (19)	−0.0102 (18)
C21	0.111 (5)	0.131 (7)	0.088 (5)	−0.003 (5)	−0.016 (4)	−0.068 (5)
C22	0.194 (10)	0.100 (6)	0.072 (4)	0.064 (6)	−0.030 (5)	−0.038 (4)
C23	0.169 (9)	0.162 (8)	0.044 (3)	−0.053 (7)	0.024 (4)	−0.020 (4)
C21'	0.13 (3)	0.12 (3)	0.13 (3)	−0.006 (19)	−0.007 (19)	−0.019 (19)
C22'	0.11 (2)	0.10 (2)	0.09 (2)	0.019 (17)	−0.012 (17)	−0.026 (18)
C23'	0.13 (3)	0.11 (3)	0.11 (2)	0.003 (19)	−0.011 (19)	−0.014 (18)
N3	0.0484 (16)	0.0451 (15)	0.0363 (13)	0.0073 (12)	−0.0044 (12)	0.0032 (12)
N4	0.0514 (15)	0.0418 (14)	0.0319 (11)	0.0064 (12)	−0.0039 (12)	−0.0008 (11)
O1	0.0715 (19)	0.086 (2)	0.0412 (12)	0.0343 (17)	0.0099 (13)	0.0085 (14)
O2	0.0519 (12)	0.0519 (12)	0.0422 (17)	−0.0066 (17)	0.0024 (11)	−0.0024 (11)

### Geometric parameters (Å, °)

**Table:**

C1—C9	1.390 (5)	C17—C18	1.384 (6)
C1—C6	1.411 (5)	C17—C20	1.545 (5)
C1—C2	1.437 (4)	C18—C19	1.387 (5)
C2—N3	1.360 (4)	C18—H18	0.9300
C2—C3	1.366 (4)	C19—H19	0.9300
C3—N4	1.383 (4)	C20—C22	1.511 (6)
C3—C4	1.431 (5)	C20—C23	1.516 (6)
C4—C12	1.403 (5)	C20—C21	1.522 (6)
C4—C5	1.418 (4)	C20—C21'	1.524 (10)
C5—N2	1.346 (4)	C20—C23'	1.527 (10)
C5—C6	1.465 (5)	C20—C22'	1.532 (10)
C6—N1	1.354 (4)	C21—H21A	0.9600
N1—C7	1.312 (6)	C21—H21B	0.9600
C7—C8	1.381 (7)	C21—H21C	0.9600
C7—H7	0.9300	C22—H22A	0.9600
C8—C9	1.372 (6)	C22—H22B	0.9600
C8—H8	0.9300	C22—H22C	0.9600
C9—H9	0.9300	C23—H23A	0.9600
N2—C10	1.316 (5)	C23—H23B	0.9600
C10—C11	1.382 (5)	C23—H23C	0.9600
C10—H10	0.9300	C21'—H21D	0.9600
C11—C12	1.367 (5)	C21'—H21E	0.9600
C11—H11	0.9300	C21'—H21F	0.9600
C12—H12	0.9300	C22'—H22D	0.9600
C13—N4	1.323 (4)	C22'—H22E	0.9600
C13—N3	1.359 (4)	C22'—H22F	0.9600
C13—C14	1.472 (4)	C23'—H23D	0.9600
C14—C19	1.383 (5)	C23'—H23E	0.9600
C14—C15	1.389 (5)	C23'—H23F	0.9600
C15—C16	1.381 (5)	N3—H3	0.86 (3)
C15—H15	0.9300	O1—H1A	0.83 (3)
C16—C17	1.383 (5)	O1—H1B	0.83 (3)
C16—H16	0.9300	O2—H2A	0.82 (3)
			
C9—C1—C6	118.0 (3)	C19—C18—H18	119.0
C9—C1—C2	125.5 (3)	C14—C19—C18	120.5 (4)
C6—C1—C2	116.4 (3)	C14—C19—H19	119.7
N3—C2—C3	106.4 (3)	C18—C19—H19	119.7
N3—C2—C1	130.4 (3)	C22—C20—C23	110.0 (5)
C3—C2—C1	123.2 (3)	C22—C20—C21	108.5 (5)
C2—C3—N4	110.1 (3)	C23—C20—C21	107.6 (5)
C2—C3—C4	121.7 (3)	C23—C20—C21'	135 (2)
N4—C3—C4	128.2 (3)	C22—C20—C23'	136.5 (19)
C12—C4—C5	117.4 (3)	C21'—C20—C23'	107.3 (10)
C12—C4—C3	125.1 (3)	C21—C20—C22'	136.1 (18)
C5—C4—C3	117.5 (3)	C21'—C20—C22'	107.1 (9)
N2—C5—C4	121.7 (3)	C23'—C20—C22'	106.7 (9)
N2—C5—C6	118.1 (3)	C22—C20—C17	110.9 (3)
C4—C5—C6	120.2 (3)	C23—C20—C17	108.1 (4)
N1—C6—C1	122.2 (3)	C21—C20—C17	111.7 (4)
N1—C6—C5	116.9 (3)	C21'—C20—C17	115 (2)
C1—C6—C5	121.0 (3)	C23'—C20—C17	110.1 (19)
C7—N1—C6	117.4 (4)	C22'—C20—C17	110.7 (17)
N1—C7—C8	124.8 (4)	C20—C21—H21A	109.5
N1—C7—H7	117.6	C20—C21—H21B	109.5
C8—C7—H7	117.6	C20—C21—H21C	109.5
C9—C8—C7	118.5 (4)	C20—C22—H22A	109.5
C9—C8—H8	120.8	C20—C22—H22B	109.5
C7—C8—H8	120.8	C20—C22—H22C	109.5
C8—C9—C1	119.2 (4)	C20—C23—H23A	109.5
C8—C9—H9	120.4	C20—C23—H23B	109.5
C1—C9—H9	120.4	C20—C23—H23C	109.5
C10—N2—C5	118.5 (3)	C20—C21'—H21D	109.5
N2—C10—C11	124.2 (3)	C20—C21'—H21E	109.5
N2—C10—H10	117.9	H21D—C21'—H21E	109.5
C11—C10—H10	117.9	C20—C21'—H21F	109.5
C12—C11—C10	118.4 (4)	H21D—C21'—H21F	109.5
C12—C11—H11	120.8	H21E—C21'—H21F	109.5
C10—C11—H11	120.8	C20—C22'—H22D	109.5
C11—C12—C4	119.7 (3)	C20—C22'—H22E	109.5
C11—C12—H12	120.1	H22D—C22'—H22E	109.5
C4—C12—H12	120.1	C20—C22'—H22F	109.5
N4—C13—N3	112.1 (3)	H22D—C22'—H22F	109.5
N4—C13—C14	125.0 (3)	H22E—C22'—H22F	109.5
N3—C13—C14	122.8 (3)	C20—C23'—H23D	109.5
C19—C14—C15	117.7 (3)	C20—C23'—H23E	109.5
C19—C14—C13	121.8 (3)	H23D—C23'—H23E	109.5
C15—C14—C13	120.4 (3)	C20—C23'—H23F	109.5
C16—C15—C14	121.3 (3)	H23D—C23'—H23F	109.5
C16—C15—H15	119.4	H23E—C23'—H23F	109.5
C14—C15—H15	119.4	C13—N3—C2	106.9 (3)
C15—C16—C17	121.5 (3)	C13—N3—H3	123 (3)
C15—C16—H16	119.3	C2—N3—H3	128 (3)
C17—C16—H16	119.3	C13—N4—C3	104.5 (3)
C16—C17—C18	117.0 (3)	C13—N4—H2A	124.9 (13)
C16—C17—C20	121.9 (4)	C3—N4—H2A	121.2 (13)
C18—C17—C20	121.1 (3)	H3—O1—H1A	107 (4)
C17—C18—C19	122.1 (4)	H3—O1—H1B	128 (4)
C17—C18—H18	119.0	H1A—O1—H1B	109 (4)

### Hydrogen-bond geometry (Å, °)

**Table:**

*D*—H···*A*	*D*—H	H···*A*	*D*···*A*	*D*—H···*A*
O1—H1A···N1^i^	0.83 (3)	2.45 (3)	3.193 (5)	151 (5)
O1—H1A···N2^i^	0.83 (3)	2.19 (4)	2.853 (4)	137 (5)
O1—H1B···O2^ii^	0.83 (3)	2.07 (2)	2.878 (3)	168 (5)
O2—H2A···N4	0.82 (3)	2.15 (2)	2.914 (4)	156 (5)
N3—H3···O1	0.86 (3)	1.90 (3)	2.754 (4)	176 (4)
C12—H12···N4^iii^	0.93	2.53	3.346 (4)	147

Symmetry codes: (i) −*y*+3/2, *x*−1/2, *z*−1/4; (ii) *y*+1/2, −*x*+1/2, *z*+1/4; (iii) *y*, *x*, −*z*+2.

## References

[bb1] Bian, Z. Q., Wang, K. Z. & Jin, L. P. (2002). *Polyhedron*, **21**, 313–319.

[bb2] Bruker (1997). *SMART* Bruker AXS Inc., Madison, Wisconsin, USA.

[bb3] Bruker (1999). *SAINT* Bruker AXS Inc., Madison, Wisconsin, USA.

[bb4] Cardinaels, T., Ramaekers, J., Guillon, D., Donnio, B. & Binnemans, K. (2005). *J. Am. Chem. Soc.* **127**, 17602–17603. 10.1021/ja056664w 16351079

[bb5] Janiak, C. (2000). *J. Chem. Soc. Dalton Trans.* pp. 3885–3896.

[bb6] Liu, Y., Duan, Z. Y., Zhang, H. Y., Jiang, X. L. & Han, J. R. (2005). *J. Org. Chem.* **70**, 1450–1455. 10.1021/jo047968o 15704983

[bb7] Sheldrick, G. M. (2008). *Acta Cryst.* A**64**, 112–122. 10.1107/S0108767307043930 18156677

[bb8] Spek, A. L. (2009). *Acta Cryst.* D**65**, 148–155. 10.1107/S090744490804362X PMC2631630 19171970

[bb9] Wu, J. Z., Yang, G., Chen, S., Ji, L. N., Zhou, J. Y. & Xu, Y. (1998). *Inorg. Chim. Acta*, **283**, 17–23.

